# Supplementary material for: What Factors Affect Binocular Summation?
Source: Brain Sci. 2024 Nov 28;14(12):1205. doi: 10.3390/brainsci14121205 (PMC11674417; doi:10.3390/brainsci14121205)
Supplement: Supplementary file 1 [file brainsci-14-01205-s001.zip › brainsci-3298549-supplementary.pdf]

Supplementary Material

Table S1. Pre-clinical optometric tests for normal subjects*. XP Exophoria, EP Esophoria.* ***(A).*** *Monocular and binocular visual acuity were measured by ETDRS (log-MAR chart).* ***(B)****. Refractive corrections.* ***(C).*** *Stereoscopic vision exam.* ***(D)****. The dominant eye was evaluated using the forcing monocular vision technique.* ***(E).*** *Binocular deviations were determined using the 'cover test' method; occlusion of the good eye revealed the type and direction of Tropia. The angle of phoria was measured using the alternating cover test (ACT).*

Participant	Dominant Eye	RANDOT STEREO TEST (Seconds of Arc)	VA (log MAR)			Correction		Cover test [Δ D]	
			RE	LE	BIN	RE	LE	Distance	Near
1	RE	40	0	0	-0.1	PL	PL	Ortho	Ortho
2	RE	40	0	0	-0.1	PL	PL	Ortho	Ortho
3	RE	40	0	0	-0.1	PL	PL	Ortho	Ortho
4	RE	20	0	0	-0.1	PL	PL	Ortho	3 XP
5	RE	20	0	0	-0.1	PL	PL	Ortho	Ortho
6	RE	20	0	0	-0.1	-0.75	-0.75	Ortho	Ortho
7	RE	20	0	0	0	PL	PL	Ortho	Ortho
8	RE	20	0	0	-0.1	PL	PL	Ortho	Ortho
9	LE	20	0	0	-0.1	PL	PL	Ortho	Ortho
10	RE	20	0	0	-0.1	PL	PL	Ortho	Ortho

Results

Experiment 1B: Longer to shorter presentation times (non-mixed between eyes)

We performed a three-way ANOVA test to determine the effect of the presentation time, the stimulus condition, and the group {the monocular or binocular presentation} on the contrast detection threshold.

There was a significant effect of the presentation time (F (3,156) =146.10, p=0.00), the stimulus condition (F (4,156) =36.22, p=0.00), and the group {the monocular or binocular presentation} (F (1,156) =165.93, p=0.00) on the contrast detection threshold. There was no significant interaction between the effect of the presentation time and the stimulus condition (F (12,156) =1.34, p=0.20). However, there was a significant interaction between the effect of the stimulus condition and the group (F (4,156) =10.35, p=0.00), and there was a significant interaction between the effect of the presentation time and the group (F (3,156) =3.22, p=0.02). Specifically, there was a significant difference between the monocular and binocular contrast detection threshold under the single target condition at presentation times of 200, 120, 80, and 40 ms (p=0.001, p=0.0003, p=0.0002, p=0.00 using Tukey's post-hoc analysis, respectively), which could be explained by the BS effect. However, there was no significant interaction

between the effect of the presentation time, the stimuli condition, and the group (F (12,156) =0.38, p=0.97).

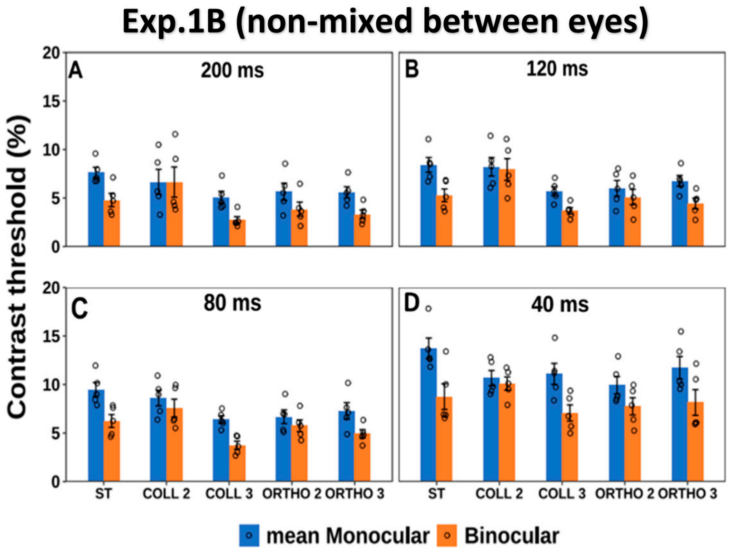

Figure S1: ***The mean Monocular and Binocular contrast detection thresholds according to presentation times of 40, 80, 120, and 200 (ms) using the LM paradigm.*** *The Single Target (ST), Collinear configuration (COLL), and Orthogonal configuration (ORTHO) with target–flanker separations of 2&3λ for each configuration.* ***A.*** *200 ms.* ***B.*** *120 ms.* ***C.*** *80 ms.* ***D.*** *40 ms. N=5, Error bars represent the standard error of the mean (SEM). Each dot represents an individual participant.*

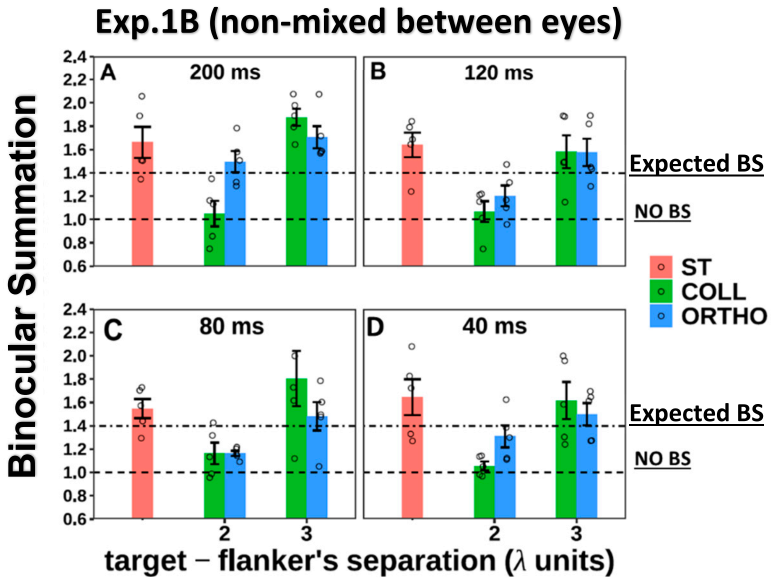

Figure S2: ***Binocular summation factor (monocular/binocular contrast detection threshold ratio) according to presentation times of 40, 80, 120, and 200 (ms) using the LM paradigm****. Single Target (ST), Collinear configuration (COLL), and Orthogonal configuration (ORTHO) with target–flanker separations of 2&3λ for each configuration.* ***A.*** *200 (ms).* ***B.*** *120 (ms).* ***C.*** *80 (ms).* ***D.*** *40 (ms). N=5, Error bars represent the standard error of the mean (SEM). Each dot represents an individual participant. The 1.4 dashed line represents the expected binocular summation (BS), whereas the 1 dashed line represents the absence of a BS effect.*

Contrast detection threshold as a function of the presentation time

Exp.1A (mixed between eyes)

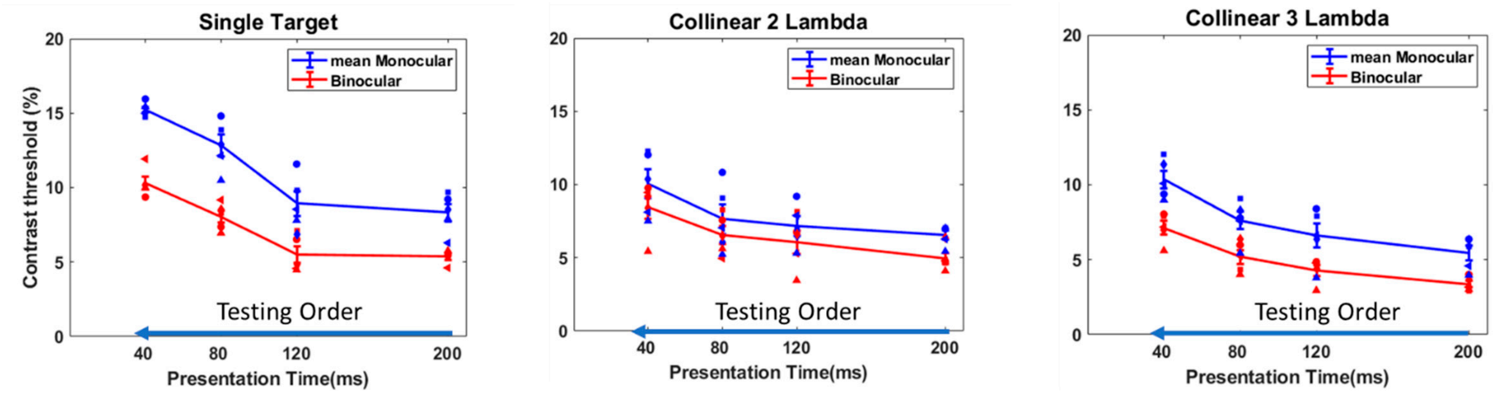

Figure S3:* The mean Monocular and Binocular contrast detection thresholds as a function of the presentation times of 40, 80,120, and 200 (ms) for experiment (1.A): longer to shorter presentation times (mixed between eyes)** *for isolated stimuli vs. stimuli with context at 4 different presentation times. N=5,* ***(each different shape represents an individual participant)*. Error bars represent the standard error of the mean (SEM). The blue arrow points to the left from longer to shorter presentation times that describe the testing order.*

Exp.1B (non-mixed between eyes)

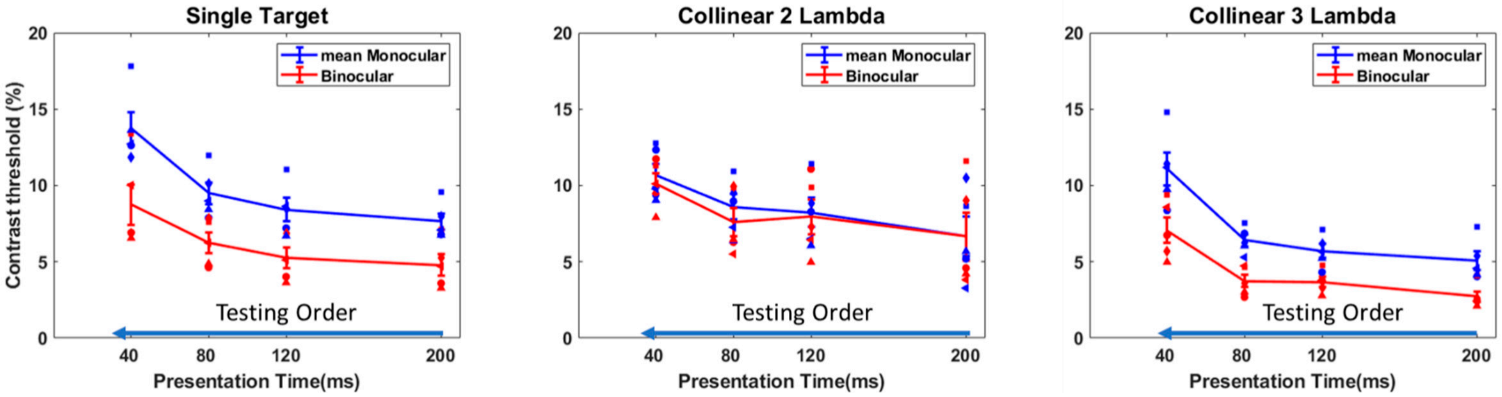

Figure S4:* The mean Monocular and Binocular contrast detection thresholds as a function of the presentation times of 40, 80,120, and 200 (ms) for experiment (1.B): longer to shorter presentation times (non-mixed between eyes)** *for isolated stimuli vs. stimuli with context at 4 different presentation times. N=5,* ***(each different shape represents an individual participant)*. Error bars represent the standard error of the mean (SEM). The blue arrow points to the left from longer to shorter presentation times that describe the testing order.*

Statistical information

Table S2. *Statistical comparison between the* ***right & left eye for contrast detection thresholds*** *under each condition throughout each presentation time during the different experiments. The evaluation was done using post-hoc analysis after 3-way ANOVA. The Single Target (ST), Collinear configuration (COLL), and Orthogonal configuration (ORTHO) with target–flanker separations of 2&3λ for each configuration.* ***Exp. 1A (mixed between eyes) and Exp. 1B (non-mixed between eyes).***

Presentation Time(ms)	Condition	Exp.1A	Exp.1B
**40**	ST	p=0.74	p=1
	COLL2	p=1	p=1
	COLL3	p=0.57	p=0.17
	ORTHO2	p=1	p=1
	ORTHO3	p=0.35	p=0.95
**80**	ST	p=0.58	p=1
	COLL2	p=1	p=1
	COLL3	p=1	p=1
	ORTHO2	p=1	p=0.71
	ORTHO3	p=0.96	p=1
**120**	ST	p=0.85	p=1
	COLL2	p=1	p=0.82
	COLL3	p=1	p=1
	ORTHO2	p=1	p=1
	ORTHO3	p=1	p=1
**200**	ST	p=1	p=1
	COLL2	p=1	p=1
	COLL3	p=0.62	p=0.98
	ORTHO2	p=1	p=1
	ORTHO3	p=0.93	p=0.94

Table S3. *Statistical comparison between the* ***mean Monocular & Binocular presentations for contrast detection thresholds*** *under each condition throughout each presentation time during the different experiments. The evaluation was done using post-hoc analysis after 3-way ANOVA. The Single Target (ST), Collinear configuration (COLL), and Orthogonal configuration (ORTHO) with target–flanker separations of 2&3λ for each configuration.* ***Exp. 1A (mixed between eyes) and Exp. 1B (non-mixed between eyes).***

Presentation Time(ms)	Condition	Exp.1A	Exp.1B
40	ST	p=0*	p=0*
	COLL2	p=0.002*	p=0.71
	COLL3	p=0*	p=0*
	ORTHO2	p=0.0002*	p=0.02*
	ORTHO3	p=0*	p=0*
80	ST	p=0*	p=0.0002*
	COLL2	p=0.56	p=0.32
	COLL3	p=0.01*	p=0.002*
	ORTHO2	p=0.003*	p=0.40
	ORTHO3	p=0.005*	p=0.008*
120	ST	p=0.0006*	p=0.0003*
	COLL2	p=0.3	p=0.93
	COLL3	p=0.03*	p=0.02*
	ORTHO2	p=0.17	p=0.42
	ORTHO3	p=0.02*	p=0.009*
200	ST	p=0.002*	p=0.001*
	COLL2	p=0.1	p=1
	COLL3	p=0.07	p=0.008*
	ORTHO2	P=0.23	p=0.05*
	ORTHO3	p=0.16	p=0.01*

Table S4. *Statistical comparison between the* ***BS ratio of the different conditions*** *throughout each presentation time during the different experiments. The evaluation was done using post-hoc analysis after 2-way ANOVA. In each row the two parameters represent the two conditions with which the statistical analysis is compared. The Single Target (ST), Collinear configuration (COLL), and Orthogonal configuration (ORTHO) with target–flanker separations of 2&3λ for each configuration.* ***Exp. 1A (mixed between eyes) and Exp. 1B (non-mixed between eyes).***

Condition	Exp.1A	Exp.1B
ST-COLL2	p=0*	p=0*
ST-COLL3	p=0.96	p=0.89
COLL2-COLL3	p=0.0003*	p=0*
ORTHO2-COLL2	p=0.0065*	p=0.01*
ORTHO3-COLL3	p=0.73	p=0.51

Table S5. *Statistical comparison between the* ***mean Monocular & Binocular collinear facilitation (threshold elevation)*** *at target-flanker separations of 2&3λ throughout each presentation time during the different experiments. The evaluation was done using a post-hoc analysis after 2-way ANOVA. The statistical analysis is compared between the mean monocular and binocular for each condition, which is presented in each row for Collinear configuration (COLL) at target–flanker separations of 2&3λ.* ***Exp. 1A (mixed between eyes) and Exp. 1B (non-mixed between eyes).***

Presentation Time(ms)	Condition	Exp.1A	Exp.1B
40	COLL2	p=0.99	p=0.04*
	COLL3	p=0.99	p=1
80	COLL2	p=0.1	p=0.30
	COLL3	p=0.99	p=1
120	COLL2	p=0.71	p=0.04*
	COLL3	p=0.99	p=1
200	COLL2	p=0.99	p=0.02*
	COLL3	p=0.99	p=1

Table S6. *Statistical comparison between the* ***Binocular collinear facilitation (threshold elevation)*** *at target-flanker separations of 2&3λ throughout each presentation time during the different experiments. The evaluation was done using a post-hoc analysis after 2-way ANOVA. The statistical analysis compared the binocular collinear facilitation (the threshold elevation) for collinear configuration (COLL) at target–flanker separations of 2&3λ, which is presented in each row.* ***Exp. 1A (mixed between eyes) and Exp. 1B (non-mixed between eyes).***

Presentation Time(ms)	Condition	Exp.1A	Exp.1B
40	COLL2-COLL3	p=0.83	p=0.09
80	COLL2-COLL3	p=0.33	p=0.0001*
120	COLL2-COLL3	p=0.07	p=0*
200	COLL2-COLL3	p=0.03*	p=0*
